# Lung Function and Safety Outcomes in Patients With Moderate-to-Severe COPD Treated With Ensifentrine: A Pooled Analysis of the ENHANCE Trials

**DOI:** 10.1016/j.chpulm.2025.100210

**Published:** 2025-09-02

**Authors:** Diego J. Maselli, Jessica Bon, Tara Rheault, Amy Dixon, Daniel Reyner, Kathleen Rickard, Michael G. Lester

**Affiliations:** a Division of Pulmonary Diseases & Critical Care, University of Texas Health, South Texas Veterans Health Care System, San Antonio, TX; b Department of Pulmonary, Critical Care, Allergy, and Immunologic Diseases, Wake Forest University School of Medicine, Winston-Salem, NC; c Verona Pharma plc, Raleigh, NC; d Department of Pulmonary and Critical Care Medicine, Vanderbilt Medical Center, Nashville, TN

**Keywords:** COPD, ensifentrine, lung function, phosphodiesterase inhibitor, pooled analysis, safety

## Abstract

**Background:**

Despite the use of maintenance therapies, many patients with COPD continue to experience persistent symptoms and impaired lung function. Ensifentrine is a novel, first-in-class selective dual inhibitor of phosphodiesterase (PDE) 3 and PDE4 with demonstrated nonsteroidal antiinflammatory activity and bronchodilatory effects.

**Research Question:**

Does ensifentrine improve lung function among patients with COPD?

**Study Design and Methods:**

This prespecified, pooled analysis of the phase 3, multicenter, randomized, double-anonymized, placebo-controlled Ensifentrine as a Novel Inhaled Nebulized COPD Therapy (ENHANCE-1 [NCT04535986] and ENHANCE-2 [NCT04542057]) trials evaluated the effect of ensifentrine on lung function and safety outcomes. The trials included patients 40 to 80 years of age with symptomatic moderate-to-severe COPD who received 3 mg twice-daily ensifentrine or placebo for 24 weeks.

**Results:**

The pooled analysis included 975 patients treated with ensifentrine and 574 patients who received placebo. Ensifentrine significantly improved average FEV_1_ area under the curve over 12 hours at week 12 from baseline vs placebo (least squares [LS] mean difference, 90 mL). Rapid improvements in peak FEV_1_ were observed with ensifentrine treatment as early as day 1 (LS mean difference, 154 mL), which were sustained through week 24 compared with placebo (LS mean difference, 135 mL) (*P* < .0001 for all time points). Ensifentrine also demonstrated significant improvements across all other lung function parameters, including average FEV_1_ area under the curve from administration time to 4 hours (LS mean difference, 137 mL at week 12; *P* < .0001 for all time points), FEV_1_ area under the curve from 6 hours to 12 hours (LS mean difference, 59 mL at week 12; *P* < .0001), morning trough FEV_1_ (LS mean difference, 42 mL at week 12; *P* < .001), and evening trough FEV_1_ (LS mean difference, 56 mL at week 12; *P* < .0001). Ensifentrine was well tolerated, with adverse event rates similar to placebo.

**Interpretation:**

Ensifentrine demonstrated early, sustained, and clinically significant improvements in lung function in a broad population and across all subgroups of patients with symptomatic moderate-to-severe COPD.

**Take-Home Points**

**Study Question:** Does ensifentrine improve lung function among patients with COPD?

**Results:** In this pooled analysis of the phase 3 Ensifentrine as a Novel Inhaled Nebulized COPD Therapy (ENHANCE) trials, ensifentrine demonstrated significant improvements in lung function in patients with moderate-to-severe COPD for all evaluated parameters compared with placebo and was well tolerated, with a pooled safety profile consistent with the individual trials and similar to placebo.

**Interpretation:** These findings support the therapeutic potential of ensifentrine in a broad population of patients with moderate-to-severe COPD.

COPD is a progressive respiratory condition characterized by chronic inflammation. This inflammation leads to airway structural changes and lung parenchymal destruction, ultimately causing airflow obstruction that contributes to persistent symptoms, exacerbations, and a decline in lung function.^1^ As the foundation of pharmacologic therapy for most patients with clinically significant COPD, inhaled bronchodilators reduce airway obstruction and increase airflow, thereby increasing FEV_1_ and/or changing other spirometric variables.^1^ Conventional long-acting bronchodilators include long-acting muscarinic antagonists (LAMAs) and long-acting beta-agonists (LABAs), which exert their effects by inducing airway smooth muscle relaxation.^2^ Despite these therapies, many patients with COPD continue to experience significant dyspnea, which negatively affects quality of life. Patients with COPD may also experience exacerbations due to various factors, including respiratory infections (viral or bacterial), environmental pollutants, or other inflammatory insults, which can lead to further decline in lung function and increased morbidity.^1^^,^^3^, ^4^, ^5^ Given the progressive nature of COPD, additional therapeutic options with alternative or complementary mechanisms are needed to further improve lung function and reduce symptoms.

Second messenger molecules cyclic adenosine monophosphate (cAMP) and cyclic guanosine monophosphate (cGMP) play a crucial role in regulating intracellular processes in the lungs, where they regulate smooth muscle tone, inflammatory processes, and mucociliary clearance.^6^^,^^7^ Phosphodiesterase (PDE) enzymes regulate cAMP and cGMP activity. In the lungs, PDE3 and PDE4 hydrolyze these cyclic nucleotides to their inactive form, making selective PDE inhibitors a promising mechanism for bronchodilation, antiinflammatory activity, and increased ciliary function for obstructive and inflammatory respiratory diseases.^6^ PDE3 modulates both cAMP and cGMP in airway smooth muscle, directly influencing bronchial tone and promoting bronchodilation.^8^, ^9^, ^10^ PDE4 primarily regulates cAMP and is integral to inflammatory cell activation and migration.^11^ It also contributes to cystic fibrosis transmembrane conductance regulator stimulation in bronchial epithelial cells, enhancing ciliary function.^12^, ^13^, ^14^, ^15^, ^16^ Dual inhibition of PDE3 and PDE4 has demonstrated synergistic effects on bronchodilation and anti-inflammatory activity compared with inhibition of either enzyme alone.^17^, ^18^, ^19^

Ensifentrine is a novel, first-in-class, selective dual inhibitor of PDE3 and PDE4 with demonstrated bronchodilatory and nonsteroidal antiinflammatory effects.^11^^,^^20^, ^21^, ^22^, ^23^, ^24^, ^25^, ^26^, ^27^, ^28^ In 2 phase 3 clinical trials, Ensifentrine as a Novel Inhaled Nebulized COPD Therapy (ENHANCE-1 [NCT04535986] and ENHANCE-2 [NCT04542057]), ensifentrine was shown to produce early, sustained, and clinically meaningful improvements in lung function, dyspnea, COPD symptoms, and quality of life, and demonstrated a favorable safety profile in symptomatic patients with moderate-to-severe COPD.^29^ Here, we report on the effect of nebulized ensifentrine 3 mg administered twice daily over 24 weeks on lung function and safety outcomes using pooled data from the ENHANCE-1 and ENHANCE-2 trials, including an analysis of the effect of ensifentrine in clinically relevant patient subgroups.

## Study Design and Methods

### Study Design

This analysis used pooled data from the ENHANCE-1 and -2 trials modified intent-to-treat (mITT) populations, which included all randomized patients who received ≥ 1 dose of study medication (ensifentrine or placebo). The ENHANCE program included 2 phase 3, global, multicenter, randomized, double-anonymized, parallel-group, placebo-controlled trials in patients with symptomatic moderate-to-severe COPD. The trial designs have been described in detail previously.^29^

Patients with moderate-to-severe COPD were enrolled and permitted to be on no maintenance therapy or stable background maintenance therapy with either an LAMA or LABA, with or without an inhaled corticosteroid (ICS). All patients were provided with albuterol or salbutamol for rescue therapy as needed during the study. Patients were randomized 5:3 to receive twice-daily ensifentrine (3 mg) or placebo via standard jet nebulizer for 24 weeks. A subset of patients in the ENHANCE-1 trial were randomized 3:1 to receive treatment for 48 weeks. In ENHANCE-1, randomization was stratified by treatment duration (24 or 48 weeks), stable background maintenance therapy with LAMA or LABA (yes or no), and smoking status (active or prior). In ENHANCE-2, randomization was stratified by stable background maintenance therapy with LAMA or LABA (yes or no) and smoking status (active or prior).

### Patients

Inclusion and exclusion criteria for the ENHANCE trials have been described previously.^29^ Briefly, eligible patients aged 40 to 80 years had a confirmed diagnosis of moderate-to-severe COPD, with a postbronchodilator FEV_1_/FVC ratio < 0.7 and an FEV_1_ of 30% to 70% predicted. Patients were required to be symptomatic, with a score ≥ 2 on the modified Medical Research Council Dyspnea Scale^30^ and a smoking history of ≥ 10 pack-years. Key exclusion criteria were a diagnosis of asthma; history of life-threatening COPD; recent hospitalizations due to COPD, pneumonia, or COVID-19 within 12 weeks of screening; and a COPD exacerbation requiring oral or IV steroids within 3 months of screening. The use of LABA/LAMA or triple (LABA/LAMA/ICS) therapy was not permitted during the trial.

### Efficacy and Safety Evaluations

**e-Figure S1:**
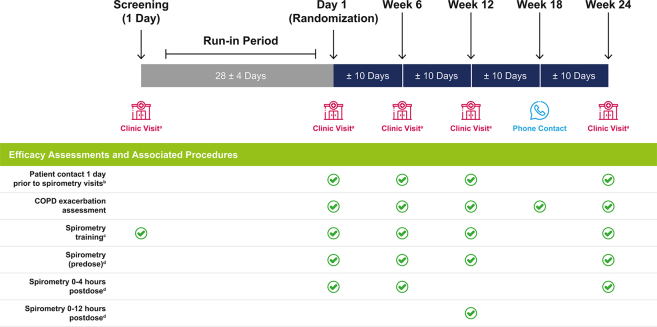


Lung function and safety were assessed over 24 weeks in both ENHANCE trials, and safety was assessed over 48 weeks in the 48-week subset of ENHANCE-1; the safety results from this subset have been published previously.^29^ The primary end point of the ENHANCE trials was the average change from baseline in FEV_1_ area under the curve during a 12-hour dosing interval (AUC_0-12h_) at week 12. Secondary lung function end points included change from baseline FEV_1_ to peak FEV_1_ (measured over 4 hours) on day 1, week 6, week 12, and week 24; change from baseline FEV_1_ to average FEV_1_ AUC_0-4h_ on day 1, week 6, week 12, and week 24; change from baseline FEV_1_ to average FEV_1_ area under the curve from 6 hours to 12 hours (AUC_6-12h_) at week 12; change from baseline FEV_1_ to morning trough FEV_1_ at week 12; and change from baseline FEV_1_ to evening trough FEV_1_ at week 12 (e-Fig 1).

**Figure 1:**
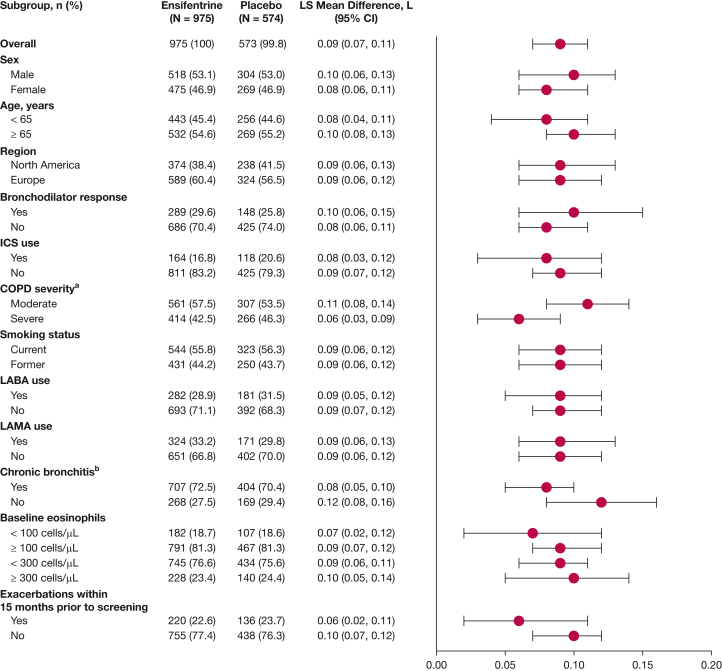
Subgroup analysis of average FEV_1_ Area under the curve for the first 12 hours following administration (AUC_0-12h_) at week 12 in the pooled Ensifentrine as a Novel Inhaled Nebulized COPD Therapy (ENHANCE) modified intent-to-treat population. ^a^COPD severity was classified as mild (postbronchodilator dose FEV_1_ ≥ 80% predicted), moderate (50% ≤ baseline FEV_1_ < 80% predicted), severe (30% ≤ baseline FEV_1_ < 50% predicted), or very severe (baseline FEV_1_ < 30% predicted). Two patients with mild COPD and 4 patients with very severe COPD were randomized in error but were allowed to continue in the trial in the moderate and severe COPD groups, respectively. ^b^Chronic bronchitis was defined as regular production of sputum for ≥ 3 mo in 2 consecutive years in the absence of other explanatory conditions. ICS = inhaled corticosteroid; LABA = long-acting beta-agonist; LAMA = long-acting muscarinic antagonist; LS = least squares.

Lung function measures were evaluated using centralized spirometry equipment and blinded central over-reads.^29^ Before the initiation of any spirometry assessment, patients in the background maintenance LAMA or LABA therapy stratum were required to withhold twice-daily maintenance LAMA or LABA for 24 hours and once-daily maintenance LAMA or LABA for 48 hours. In addition, all patients were required to withhold short-acting rescue medication for ≥ 4 hours before initiation of any spirometry.

The effect of ensifentrine was also examined in clinically relevant subgroups, including age (< 65 years or ≥ 65 years), sex (male or female), smoking status (active or prior), background medication use, including LABA or LAMA (yes or no), or ICS (yes or no). Clinical characteristics included baseline bronchodilator responsiveness (yes or no), history of chronic bronchitis (yes or no), severity of bronchial obstruction (moderate or severe COPD), baseline blood eosinophil count (≥ 100 or < 100 cells/μL and ≥ 300 or < 300 cells/μL), and prior history of exacerbations (0 or ≥ 1 in the 15 months preceding screening).

Safety outcomes were assessed in the pooled ENHANCE mITT patient population. Safety outcomes included treatment-emergent adverse events (TEAEs), electrocardiogram abnormalities, and vital signs.

### Statistical Analysis

This pooled post hoc analysis over 24 weeks was conducted as a secondary, exploratory evaluation of data from the individual ENHANCE trials that have been described previously.^29^ Average FEV_1_ AUC_0-12h_ was calculated using the trapezoidal method based on predose and postdose measurements at 30 minutes and 1, 2, 4, 6, 8, and 12 hours. Analysis conventions were followed as previously described for ENHANCE-1 and ENHANCE-2,^29^ with an additional model term for study included into statistical models in pooled analyses. Missing data post-baseline were imputed for each study by multiple imputation under a missing at random assumption. The combined data were analyzed using analysis of covariance models with terms for study, region, treatment group, and randomization strata, with baseline FEV_1_ as a covariate. Estimates were combined across imputations using Rubin’s rules, and least squares (LS) means, LS mean treatment differences, associated 95% CIs, and *P* values obtained are presented. Safety outcomes were analyzed descriptively.

Markov chain Monte Carlo imputation was used, assuming data followed an arbitrarily missing pattern through the observed data, and the resultant data sets with a monotone missing pattern after Markov chain Monte Carlo imputation used the regression method for imputation. Tipping-point analyses were performed to examine the sensitivity of results to the missing at random assumption.

### Oversight

The ENHANCE-1 and ENHANCE-2 trials were conducted in accordance with the Declaration of Helsinki and Good Clinical Practice guidelines (ICH/CPMP/135/95). All patients provided written informed consent before participation. Trial conduct was approved by independent ethics committees or institutional review boards at each participating site. Data from 2 trial sites in ENHANCE-1 and 1 site in ENHANCE-2 were excluded from all analyses before database lock and unblinding due to noncompliance with Good Clinical Practice. Details regarding participating study sites and ethics committees have been described previously.^29^

## Results

### Patients

The pooled mITT population included 1,549 patients, with 975 randomized to ensifentrine and 574 randomized to placebo.^31^ Baseline demographic and patient characteristics were similar between the treatment groups (Table 1). The mean age of patients was 65.1 years; 54.6% of patients in the ensifentrine group and 55.2% in the placebo group were ≥ 65 years of age. In both groups, 46.9% of patients were female, and most were White (ensifentrine, 92.9%; placebo, 91.6%).

**Table 1:** Baseline Demographic and Clinical Characteristics of the Pooled ENHANCE mITT Population^a^

Characteristic	Ensifentrine (n = 975)	Placebo (n = 574)
Demographics		
Age ≥ 65 y	532 (54.6)	317 (55.2)
Female	457 (46.9)	269 (46.9)
Geographic region		
North America	374 (38.4)	238 (41.5)
Europe	589 (60.4)	325 (56.6)
Asia	12 (1.2)	11 (1.9)
Disease characteristics		
History of chronic bronchitis^b^	707 (72.5)	405 (70.6)
% predicted FEV_1_, mean (SD)	51.8 (10.6)	51.0 (10.6)
Moderate COPD^c^	561 (57.5)	307 (53.5)
Severe COPD^c^	414 (42.5)	267 (46.5)
Exacerbation within 15 mo before screening	220 (22.6)	136 (23.7)
Active tobacco use	544 (55.8)	323 (56.3)
Respiratory assessment scores, mean (SD)		
E-RS total score	13.7 (6.7)	13.3 (6.1)
SGRQ total score	49.4 (17.9)	49.1 (16.9)
BDI score	5.9 (1.2)	5.9 (1.2)
Concomitant medications	606 (62.2)	352 (61.3)
LAMA	324 (33.2)	171 (29.8)
LABA	282 (28.9)	181 (31.5)
LABA/ICS	159 (16.3)	113 (19.7)
Patients with ≥ 1 concomitant illness^d^	875 (89.7)	520 (90.6)
Vascular disorders	627 (64.3)	358 (62.4)
Hypertension	580 (59.5)	325 (56.6)
Peripheral vascular disorder	31 (3.2)	16 (2.8)
Arteriosclerosis	24 (2.5)	24 (4.2)
Gastrointestinal disorders	311 (31.9)	177 (30.8)
Gastroesophageal reflux disease	207 (21.2)	111 (19.3)
Psychiatric disorders	259 (26.6)	158 (27.5)
Depression	148 (15.2)	99 (17.2)
Anxiety	109 (11.2)	70 (12.2)
Insomnia	104 (10.7)	63 (11.0)
Cardiac disorders	245 (25.1)	157 (27.4)
Coronary artery disease	84 (8.6)	61 (10.6)
Myocardial ischemia	39 (4.0)	17 (3.0)
Atrial fibrillation	32 (3.3)	14 (2.4)
Angina pectoris	25 (2.6)	18 (3.1)

Data are presented as No. (%) or as otherwise indicated. BDI = Baseline Dyspnea Index; ENHANCE = Ensifentrine as a Novel Inhaled Nebulized COPD Therapy; E-RS = Evaluating Respiratory Symptoms in COPD; ICS = inhaled corticosteroid; LABA = long-acting beta-agonist; LAMA = long-acting muscarinic antagonist; mITT = modified intent-to-treat; SGRQ = St. George’s Respiratory questionnaire.

a mITT population is defined as randomized patients who received ≥ 1 dose of study medication (ie, ensifentrine, placebo).

b Chronic bronchitis was defined as regular production of sputum for ≥ 3 mo in 2 consecutive y in the absence of other conditions that may explain it.

c COPD severity was defined as mild (postbronchodilator dose FEV_1_ ≥ 80% predicted), moderate (50% ≤ baseline FEV_1_ < 80% predicted), severe (30% ≤ baseline FEV_1_ <50% predicted), and very severe (baseline FEV_1_ < 30% predicted). Two patients with mild COPD and 4 patients with very severe COPD were randomized in error and allowed to continue in the trial in the moderate and severe COPD groups, respectively.

d Concomitant illness refers to events ongoing or beginning after the screening visit.

At baseline, the mean postbronchodilator FEV_1_ percent predicted ± SD was 51.8% ± 10.6% in the ensifentrine group and 51.0% ± 10.6% in the placebo group. With respect to COPD severity, 57.5% and 53.5% of patients in the ensifentrine and placebo groups were classified as having moderate COPD, respectively, and 42.5% and 46.5% were classified as having severe COPD (Table 1).

### Efficacy

#### Lung Function Outcomes

Ensifentrine demonstrated significant increases vs placebo in all prespecified FEV_1_-based lung function parameters, beginning on day 1 and continuing through week 24 of the ENHANCE trials (Table 2).

**Table 2:** Lung Function Analyses in the Pooled ENHANCE Modified Intent-to-Treat Population

End Point	Outcome Measure, LS Mean Change From Baseline, mL	Study Visit	Ensifentrine (n = 975)	Placebo (n = 573)	Difference (95% CI)	*P* Value^a^
Primary	Average FEV_1_ AUC_0-12h_^b^	Week 12	55 (15.7)	−36 (16.3)	90 (69-112)	< .0001
Secondary	Peak FEV_1_ over 4 h	Day 1	222 (10.9)	68 (11.3)	154 (140-169)	< .0001
	Peak FEV_1_ over 4 h	Week 6	199 (16.5)	55 (17.3)	144 (121-167)	< .0001
	Peak FEV_1_ over 4 h	Week 12	199 (17.2)	52 (18.2)	146 (122-171)	< .0001
	Peak FEV_1_ over 4 h	Week 24	166 (18.5)	31 (19.5)	135 (108-161)	< .0001
	Average FEV_1_ AUC_0-4h_	Day 1	147 (9.2)	3 (9.7)	144 (132-157)	< .0001
	Average FEV_1_ AUC_0-4h_	Week 6	124 (15.7)	−13 (16.4)	137 (115-159)	< .0001
	Average FEV_1_ AUC_0-4h_	Week 12	121 (16.2)	−16 (16.9)	137 (115-160)	< .0001
	Average FEV_1_ AUC_0-4h_	Week 24	94 (17.5)	−27 (18.4)	121 (97-145)	< .0001
	Average FEV_1_ AUC_6-12h_^b^	Week 12	1 (17.2)	−58 (18.0)	59 (36-81)	< .0001
	Morning trough FEV_1_	Week 12	11 (16.3)	−31 (17.0)	42 (19-65)	.0003
	Evening trough FEV_1_^b^	Week 12	−21 (18.4)	−77 (18.8)	56 (32-79)	< .0001

Data are presented as mean (SE) or as otherwise indicated. AUC_0-12h_ = area under the curve for the first 12 hours followin administration; AUC_0-4h_ = area under the curve for the first 4 hours after administration; AUC_6-12h_ = area under the curve for hors 6-12 after administration; ENHANCE = Ensifentrine as a Novel Inhaled Nebulized COPD Therapy; LS = least squares.

a Based on multiple imputation analyses. *P* is for a 2-sided null hypothesis of no difference between treatment groups.

b Data based on 12-h spirometry data (FEV_1_ AUC_0-12h_, FEV_1_ evening trough, and FEV_1_ AUC_6-12h_) are only available for week 12.

##### Average FEV_1_ AUC_0-12h_

The LS mean difference in FEV_1_ AUC_0-12h_ at week 12 was 90 mL (95% CI, 69-112; *P* < .0001) from baseline compared with placebo, consistent with the individual phase 3 study results. This improvement occurred across all subgroups analyzed, with significant improvements vs placebo in each subgroup (Fig 1).

##### Peak FEV_1_

**Figure 2:**
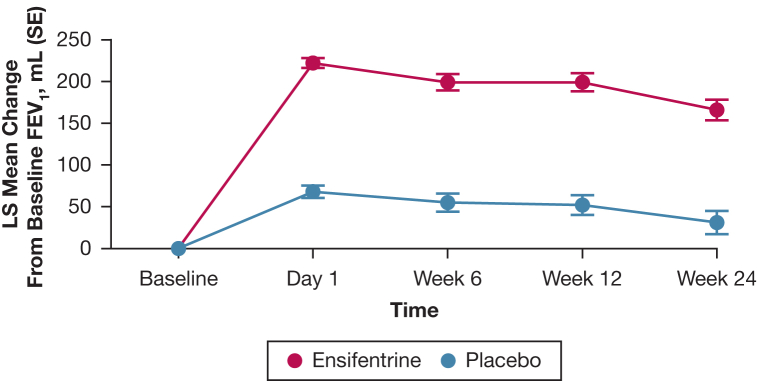
LS mean change from baseline FEV_1_ to peak FEV_1_ in the pooled ENHANCE modified intent-to-treat population. LS = least squares. ENHANCE = Ensifentrine as a Novel Inhaled Nebulized COPD Therapy.

At week 12, the LS mean difference in peak FEV_1_ in patients who received ensifentrine vs placebo was 146 mL (95% CI, 122-171; *P* < .0001) (Table 2). Ensifentrine significantly increased the LS mean change from baseline in peak FEV_1_ vs placebo, as early as day 1 and continuing through week 24 (*P* < .0001 at all time points) (Fig 2, Table 2).

##### Additional FEV_1_-Based End Points

At week 12, patients who received ensifentrine experienced an LS mean difference vs placebo in average FEV_1_ AUC_0-4h_ of 137 mL (95% CI, 115-160; *P* < .0001), in FEV_1_ AUC_6-12h_ of 59 mL (95% CI, 36-81; *P* < .0001), in morning trough FEV_1_ of 42 mL (95% CI, 19-65; *P* < .001), and in evening trough FEV_1_ of 56 mL (95% CI, 32-79; *P* < .0001). Patients who received ensifentrine had a significant improvement in LS mean average FEV_1_ AUC_0-4h_ compared with patients who received placebo as early as day 1 and continuing through week 24 (*P* < .0001 at all time points) (Table 2).

### Safety

Ensifentrine was well tolerated over 24 weeks of treatment with low rates of adverse events. A similar proportion of patients reported TEAEs in both treatment groups (ensifentrine, 36.8%; placebo, 35.9%) (Table 3). The most common (≥ 1%) TEAEs in the ensifentrine group vs placebo were back pain (1.8% vs 1.0%), hypertension (1.7% vs 0.9%), urinary tract infection (1.3% vs 1.0%), and diarrhea (1.0% vs 0.7%). The proportion of patients with serious TEAEs was also similar in the ensifentrine treatment group (6.2%) and the placebo group (6.3%). Treatment-related serious adverse events were rare, with 1 patient (0.1%) in the ensifentrine group experiencing a treatment-related serious adverse events compared with none in the placebo group. TEAEs leading to treatment withdrawal occurred in 7.6% of ensifentrine-treated patients and 8.2% of placebo-treated patients. Deaths related to TEAEs occurred in 0.6% of ensifentrine-treated patients and 0.9% of placebo-treated patients.

**Table 3:** TEAEs During 24 Weeks of Treatment in the Pooled ENHANCE Safety Population^a^

TEAE	Ensifentrine (n = 975)	Placebo (n = 574)
Any	359 (36.8)	206 (35.9)
TEAEs with ≥ 1% incidence and higher incidence in the ensifentrine group		
Back pain	18 (1.8)	6 (1.0)
Hypertension	17 (1.7)	5 (0.9)
Urinary tract infection	13 (1.3)	6 (1.0)
Diarrhea	10 (1.0)	4 (0.7)
Serious TEAEs	60 (6.2)	36 (6.3)
Treatment-related SAEs	1 (0.1)	0 (0)
TEAEs leading to treatment withdrawal	74 (7.6)	47 (8.2)
TEAEs leading to death^b^	6 (0.6)	5 (0.9)
GI TEAEs reported in > 1 patient		
GI disorders	50 (5.1)	26 (4.5)
Diarrhea	10 (1.0)	4 (0.7)
Nausea	6 (0.6)	3 (0.5)
Toothache	6 (0.6)	3 (0.5)
Gastritis	5 (0.5)	1 (0.2)
Vomiting	5 (0.5)^a^	1 (0.2)
Gastroesophageal reflux disease	3 (0.3)	4 (0.7)
Abdominal pain	3 (0.3)	1 (0.2)
Dry mouth	3 (0.3)	1 (0.2)
Hiatal hernia	2 (0.2)	3 (0.5)
Abdominal distension	2 (0.2)	1 (0.2)
Dyspepsia	2 (0.2)	1 (0.2)
Abdominal pain, upper	2 (0.2)	0 (0)
Vascular or cardiac TEAE^c^ > 1 patient		
Vascular disorders	28 (2.9)	7 (1.2)
Hypertension^c^	17 (1.7)	5 (0.9)
Hypotension	5 (0.5)	0 (0)
Cardiac disorders	23 (2.4)	17 (3.0)
Atrial fibrillation	8 (0.8)	3 (0.5)
Sinus tachycardia	3 (0.3)	0 (0)
Cardiac failure	2 (0.2)	3 (0.5)
Ventricular extrasystoles	2 (0.2)	1 (0.2)
Angina pectoris	1 (0.1)	4 (0.7)
Tachycardia	1 (0.1)	2 (0.3)
Psychiatric TEAE^c^ > 1 patient		
Psychiatric disorders	16 (1.6)	2 (0.3)
Insomnia	6 (0.6)	2 (0.3)
Anxiety	2 (0.2)	1 (0.2)
Depression-related reactions	4 (0.4)	0 (0)
Depression	1 (0.1)	0 (0)
Major depression	1 (0.1)	0 (0)
Adjustment disorder with depressed mood	2 (0.2)	0 (0)

Data are presented as No. (%). ENHANCE = Ensifentrine as a Novel Inhaled Nebulized COPD Therapy; GI = gastrointestinal; SAE = serious adverse event; TEAE = treatment-emergent adverse event.

a Safety analysis set includes all patients randomized and treated with study medication (receiving at least 1 dose).

b Ensifentrine deaths: COVID-19; COVID-19 pneumonia; adenocarcinoma, metastatic; lung neoplasm, malignant; acute left ventricular failure, and toxicity to various agents (acute cocaine toxicity). Placebo deaths: *Clostridium difficile* infection; pneumonia, bacterial; septic shock, cholangiocarcinoma; small cell lung cancer, metastatic; and shock, hemorrhagic.

c Of the patients reporting a TEAE of hypertension, 58% had a history of hypertension.

The incidence of gastrointestinal (GI) TEAEs was low and similar between treatment groups (Table 3). Diarrhea was the most frequently reported GI TEAE in both treatment groups. Of the 10 ensifentrine-treated patients who reported diarrhea, 8 reported diarrhea after treatment day 80. All GI TEAEs in the ensifentrine group were mild or moderate, and there were no reports of weight loss or abnormal loss of weight in the pooled ENHANCE safety population over 24 weeks.

Vascular events occurred in 2.9% of patients in the ensifentrine group vs 1.2% in the placebo group, and cardiac events occurred in 2.4% vs 3.0% of patients, respectively (Table 3). No episodes of ventricular tachyarrhythmias or other supraventricular tachyarrhythmias were observed in the pooled population. All cardiovascular events were mild or moderate in severity, except for 1 severe event in the ensifentrine group; the event (acute left ventricular heart failure) resulted in death. This was considered to be unrelated to study treatment due to a significant history of cardiovascular comorbidities, including a recent myocardial infarction within 6 months before participation in the study. Hypertension was reported in 1.7% of patients in the ensifentrine group and 0.9% of patients in the placebo group. Of the patients reporting a TEAE of hypertension, 58% had a history of hypertension. All events were considered mild or moderate in severity, except for 1 severe event in a patient who received ensifentrine, which the study investigator considered unrelated to the study medication. This patient continued ensifentrine after hospital discharge without recurrence.

No trends by treatment group or clinically meaningful changes from baseline or from predose to postdose were observed in any vital sign parameter during the study, including systolic and diastolic BP (e-Table 1). The proportion of patients with any markedly abnormal change relative to baseline in systolic or diastolic BP or pulse rate was low and similar across both treatment groups (≤ 2.8%) throughout the study. No clinically relevant changes in electrocardiogram parameters, including heart rate, were observed (e-Table 2).

Psychiatric disorder TEAEs were observed in 1.6% of ensifentrine-treated patients and 0.3% of placebo-treated patients (Table 3). The most commonly reported (> 1 patient in either treatment group) psychiatric TEAEs were insomnia (ensifentrine, 6 [0.6%]; placebo, 2 [0.3%]), anxiety (ensifentrine, 2 [0.2%]; placebo, 1 [0.2%]), and adjustment disorder with depressed mood (ensifentrine, 2 [0.2%]; placebo, 0). One patient who received ensifentrine in the pooled 24-week safety population experienced a suicide-related adverse event (suicide attempt), which was reported by the site investigator as unrelated to study treatment.

## Discussion

Ensifentrine, a novel dual inhibitor of PDE3 and PDE4, demonstrated early, sustained, and clinically significant improvements in lung function in a broad population and across all subgroups of patients with moderate-to-severe symptomatic COPD. The bronchodilatory effects were evident after the first dose on day 1 and sustained over 24 weeks. These data support ensifentrine as a novel bronchodilator for patients with COPD with a mechanism complementary to existing bronchodilators. Although it is well known that spirometry may not strongly correlate with patient symptomatology, postbronchodilator spirometry remains the most widely available and reproducible measure of lung function.^1^ In the current analysis, ensifentrine demonstrated lung function improvements in a population of patients, most of whom were already using concomitant bronchodilator maintenance therapy (LAMA or LABA). This adds to the previously established data on improvements in symptoms, dyspnea, quality of life, and exacerbations with ensifentrine treatment.^29^^,^^32^^,^^33^

Ensifentrine was well tolerated over 24 weeks, with low rates of TEAEs reported in both treatment groups. The incidence of serious TEAEs, TEAEs resulting in withdrawal of study medication, and TEAEs leading to death was also low in ensifentrine and placebo treatment groups.

GI adverse events are known to occur with oral PDE4 inhibitors (eg, roflumilast).^34^ In clinical trials with roflumilast, GI TEAEs (eg, diarrhea, nausea) were mainly observed in the first 4 weeks of treatment.^35^ In this pooled analysis, rates of nausea were < 1%, and rates of diarrhea were ≤ 1% in both treatment groups, suggesting improved tolerance of PDE4 inhibition with inhaled administration, which minimizes GI absorption. In contrast to oral PDE4 inhibitors, which have been associated with weight loss in clinical trials,^36^ no patients in the ensifentrine group reported weight loss or abnormal weight loss during the 24-week treatment period, further highlighting improved GI tolerability with inhaled PDE inhibition compared with oral administration. It is important to note that roflumilast is indicated to reduce the risk of COPD exacerbations in a limited population of patients with severe COPD associated with chronic bronchitis and a history of exacerbations, whereas ensifentrine is indicated for the broader maintenance treatment of COPD. A direct comparison of the effect of roflumilast vs ensifentrine on exacerbation reduction has not been conducted. Psychiatric disorders, in particular anxiety, depression, and suicidality, have been reported with oral inhibitors of PDE4,^37^ which are also known as important comorbidities in patients with COPD.^1^ Depression and anxiety were reported comorbidities in > 15% and 11% of patients enrolled in the ENHANCE program, respectively. Suicidality is a significant concern among patients with COPD, with epidemiologic studies consistently indicating an association between COPD and elevated suicide risk of approximately 2-fold compared with matched non-COPD control patients.^38^^,^^39^ A cross-sectional study of 1,190 patients with COPD (mean postbronchodilator FEV_1_, 52% predicted) reported suicide attempts in 2.5% of patients with COPD.^40^ One patient who received ensifentrine experienced a suicide-related adverse event (suicide attempt). In this pooled analysis, a small number of nonserious reports of insomnia and depression were reported at a proportionally higher rate in ensifentrine-treated patients than in patients who received placebo. Anxiety was reported with a similar incidence (0.2%) between treatment groups. However, the interpretation of these events is limited by the small number of events, nearly double exposure time in ensifentrine-treated patients compared with placebo-treated patients due to the 5:3 randomization, patient-specific confounding factors, and the fact that the study was not powered to detect differences in infrequent adverse events. In addition, nonclinical data with ensifentrine suggest that ensifentrine does not cross the blood-brain barrier (Verona Pharma, plc, unpublished data, 2025) and has maximal systemic concentrations > 500 times lower than what would be required for target engagement of PDE4 outside of the lungs. The lack of association of ensifentrine with nausea further supports a lack of activity on the central nervous system. Nevertheless, careful attention should be paid to rates of these adverse effects in future investigations for further clarification.

A limitation of this analysis is that patients who were receiving dual LAMA/LABA or triple LAMA/LABA/ICS therapy were not included in the ENHANCE program, which are the therapies recommended by the most recent Global Initiative for Chronic Obstructive Lung Disease report for patients with COPD who experience significant symptom burden or frequent exacerbations.^1^ Further studies are needed to evaluate the efficacy of ensifentrine in combination with dual bronchodilator or triple inhaled maintenance therapies to better define the optimal use cases in the treatment of COPD. Moreover, because the evening dosing was not conducted and observed in the clinic, the actual timing relative to the morning trough assessment could not be verified. Despite these limitations, the ENHANCE program provided the opportunity to evaluate the effects of ensifentrine on lung function in a cohort of well-characterized patients with COPD with a wide range of severity.

## Interpretation

In this pooled analysis of the phase 3 ENHANCE trials, ensifentrine demonstrated significant improvements in lung function in patients with moderate-to-severe COPD for all evaluated parameters compared with placebo. These improvements were observed as early as the first day of treatment and were sustained throughout the 24-week study. Ensifentrine was well tolerated, with a pooled safety profile consistent with the individual trials and similar to placebo. These findings support the therapeutic potential of ensifentrine in a broad population of patients with moderate-to-severe COPD.

## Funding/Support

Medical writing support was provided by Vivek Shah, PhD, from Citrus Health Group, Inc. (Chicago, IL), in accordance with current Good Publication Practice guidelines, and funded by Verona Pharma (Raleigh, NC).

## Financial/Nonfinancial Disclosures

The authors have reported to *CHEST Pulmonary* the following: D. J. M. is on consulting/advisory boards for GSK, AZ, Amgen, Sanofi/Regeneron, Insmed, and Verona Pharma; and reports speaker fees from GSK, AZ, Amgen, and Sanofi/Regeneron. J.B. reports grant funding from the 10.13039/100000050National Heart, Lung, and Blood Institute (NHLBI); being on consulting/advisory boards for GSK, Sanofi/Regeneron, Verona Pharma, and Chiesi; and speaker fees from GSK and Sanofi/Regeneron. T. R., A. D., D. R., and K. R. are employees of Verona Pharma. M. G. L. reports being on consulting/advisory boards for Ryme Medical, GSK and Verona Pharma.

## Supplementary Data

e-Online Data
