# Supplementary material for: Lung Function and Safety Outcomes in Patients With Moderate-to-Severe COPD Treated With Ensifentrine: A Pooled Analysis of the ENHANCE Trials
Source: CHEST Pulm. 2025 Sep 2;3(4):100210. doi: 10.1016/j.chpulm.2025.100210 (PMC13419160; doi:10.1016/j.chpulm.2025.100210)
Supplement: e-Online Data [file mmc1.docx]

**Supplementary Material**

**Lung function and safety outcomes in patients with moderate-to-severe chronic obstructive pulmonary disease treated with ensifentrine: a pooled analysis of the ENHANCE trials**

**e-Figure 1. Assessments and procedures during the 24-week ENHANCE study period**


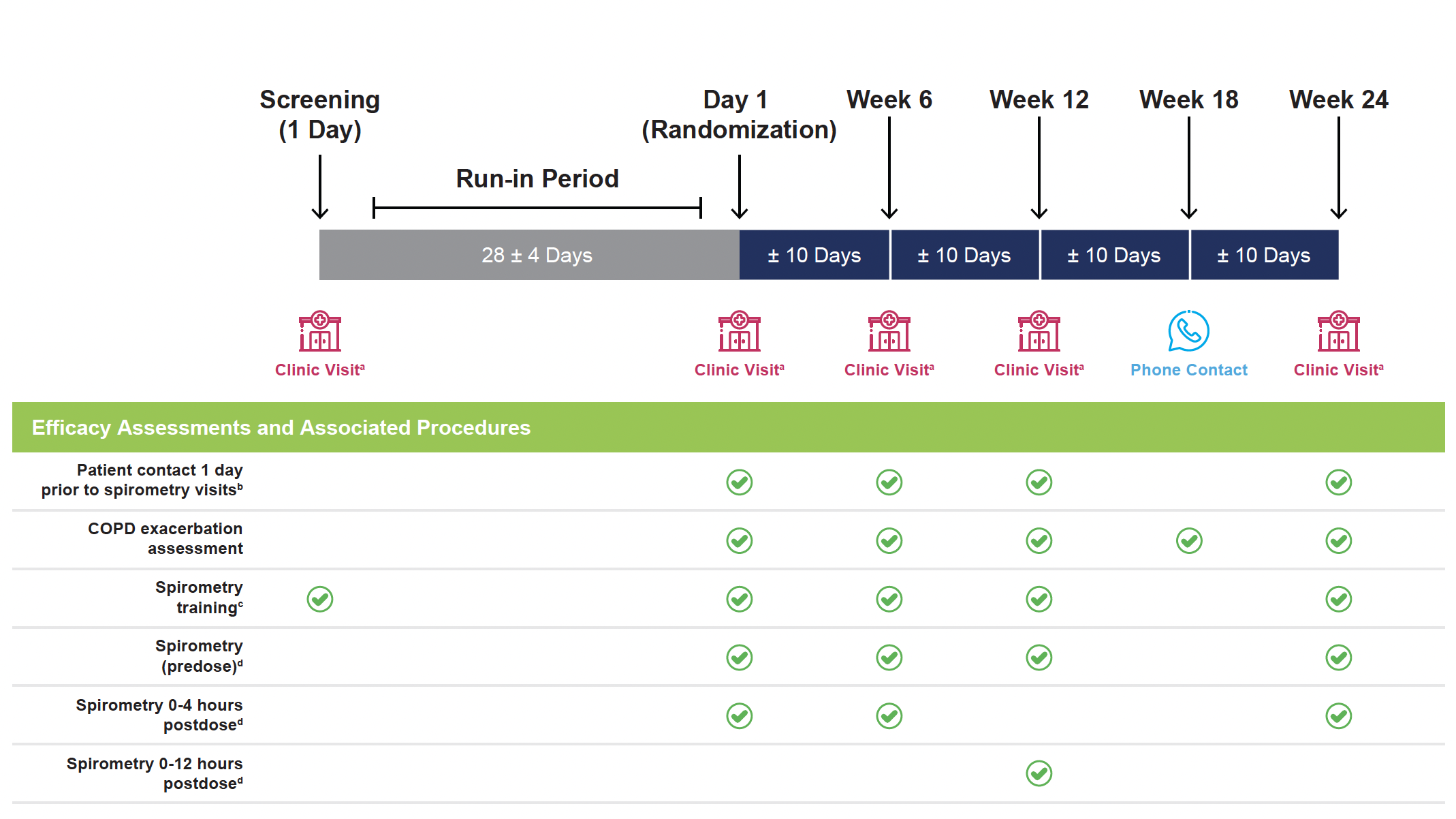


^a^No visit procedures could be completed unless all rescue medication (albuterol/salbutamol) or LAMA or LABA medication (±ICS) were withheld for the time periods defined in the study protocol. Patients were requested to withhold caffeine and smoking as per protocol.

^b^Within 4 days prior to a scheduled in-clinic visit where spirometry was scheduled and blinded study medication was administered, site staff contacted the patient (phone or text message preferred) to remind them to take their study medication (blinded) and to record the date and start and stop times of the evening dose the night before the clinic visit in their diary. Prior to clinic visits, patients were reminded to withhold their study medication on the morning of the visit as this was dosed in the clinic, to withhold rescue medication (albuterol/salbutamol) ≥4 hours prior to their visit, and to withhold LAMA or LABA medication (with or without ICS) until the end of the clinic visit.

^c^Study site staff trained patients on how to perform acceptable spirometry maneuvers before their first spirometry test, according to instructions provided by the central vendor.

^d^For each spirometry test, efforts were defined as acceptable when they included a satisfactory start and end of the test (indicated by a plateau on the volume-time curve) and were free from artifacts, such as cough, early termination, poor effort, or equipment-related issues. For FEV_1_ and FVC determinations, between 3 and 8 acceptable efforts were obtained per patient, with the largest values recorded even if they did not come from the same effort. Efficacy endpoints based on 12-hour spirometry data (FEV_1_ area under the curve during a 12-hour dosing interval [FEV_1_ AUC_0-12h_], FEV_1_ evening trough, and FEV_1_ AUC_6-12h_) were analyzed using data collected exclusively at clinic visits with 12-hour spirometry, with no windowing convention applied. Because 12-hour spirometry was only conducted at one planned time point during the study (Week 12), patients who withdrew from the study prior to this time point were encouraged to complete 12-hour spirometry at their early termination visit to minimize missing data on these endpoints. The timing of the trough FEV_1_ assessment was managed via collection of the evening dose timing in a paper diary, supported by a phone call from study personnel to remind patients about evening dose timing relative to their morning pre-dose spirometry assessment.

COPD, chronic obstructive pulmonary disease; FEV_1_, forced expiratory volume in 1 second; FVC, forced vital capacity; ICS, inhaled corticosteroid; LABA, long-acting beta-agonist; LAMA, long-acting muscarinic antagonist.

**e-Table 1. Treatment-emergent, clinically significant vital sign changes by visit over 24 weeks in the pooled ENHANCE safety population**

| ​​ Vital sign | Ensifentrine  (N=975)​ | Placebo​  (N=574)​​ |
| --- | --- | --- |
| Systolic blood pressure abnormally low,^a^ n/N (%) | | |
| Day 1 | 1/975 (0.1) | 1/574 (0.2) |
| Week 6 | 3/900 (0.3) | 5/533 (0.9) |
| Week 12​ | 6/865 (0.7) | 3/503 (0.6) |
| Week 24 | 6/815 (0.7) | 3/469 (0.6) |
| ​​Systolic blood pressure abnormally high,^b^ n/N (%) | | |
| Day 1 | 0/975 (0.0) | 1/574 (0.2) |
| Week 6 | 4/900 (0.4) | 2/533 (0.4) |
| Week 12​ | 5/865 (0.6) | 4/503 (0.8) |
| Week 24​ | 3/815 (0.4) | 1/469 (0.2) |
| ​​Diastolic blood pressure abnormally low,^a^ n/N (%) | | |
| Day 1 | 1/975 (0.1) | 0/574 (0) |
| Week 6 | 13/900 (1.4) | 11/533 (2.1) |
| Week 12 | 17/865 (2.0) | 14/503 (2.8) |
| Week 24 | 19/815 (2.3) | 7/469 (1.5) |
| Diastolic blood pressure abnormally high,^b^ n/N (%) | | |
| Day 1 | 0/975 (0) | 2/574 (0.3) |
| Week 6 | 13/900 (1.4) | 9/533 (1.7) |
| Week 12 | 11/865 (1.3) | 11/503 (2.2) |
| Week 24 | 16/815 (2.0) | 6/469 (1.3) |

^a^Abnormally low was defined by a measurement ≤90 or decrease from baseline ≥40 for systolic blood pressure or measurement ≤50 or decrease from baseline ≥20 for diastolic blood pressure.

^b^Abnormally high was defined by a measurement ≥180 or increase from baseline ≥40 for systolic blood pressure or measurement ≥105 or increase from baseline ≥20 for diastolic blood pressure.

**e-Table 2. Treatment-emergent, clinically significant ECG abnormalities by visit in the pooled ENHANCE safety population**

| ​​ ECG abnormality | Ensifentrine  (N=975)​ | Placebo​  (N=574)​​ |
| --- | --- | --- |
| Patients with 1 or more ECG abnormalities, n/N (%) | | |
| Day 1 | 126/975 (12.9) | 89/573 (15.5) |
| Week 6 | 141/897 (15.7) | 85/532 (16.0) |
| Week 12​ | 145/864 (16.8) | 93/501 (18.6) |
| Week 24 | 137/814 (16.8) | 85/468 (18.2) |
| ​​QTcF interval > 450 msec, n/N (%) | | |
| Day 1 | 54/975 (5.5) | 30/573 (5.2) |
| Week 6 | 35/896 (3.9) | 21/532 (3.9) |
| Week 12​ | 35/864 (4.1) | 21/501 (4.2) |
| Week 24​ | 30/814 (3.7) | 13/468 (2.8) |
| ​​QTcF interval > 480 msec, n/N (%) | | |
| Day 1 | 7/975 (0.7) | 0/573 (0) |
| Week 6 | 1/896 (0.1) | 1/532 (0.2) |
| Week 12 | 1/864 (0.1) | 1/501 (0.2) |
| Week 24 | 2/814 (0.2) | 0/468 (0) |
| QTcF interval > 500 msec, n/N (%) | | |
| Day 1^a^ | 2/975 (0.2) | 0/573 (0) |
| Heart rate ≥110 bpm and ≥30 bpm change from baseline, n/N (%) | | |
| Day 1 | 1/969 (0.1) | 0/569 (0) |
| Week 6 | 1/897 (0.1) | 1/531 (0.2) |
| Week 12 | 2/864 (0.2) | 1/500 (0.2) |
| Week 24 | 2/814 (0.2) | 1/467 (0.2) |

^a^No other instances of QTcF > 500 msec were noted through Week 24.

Bpm, beats per minute; ECG, electrocardiogram; QTcF, QT interval corrected for heart rate using the Fridericia method.
